# Transcriptome Analysis of Shade-Induced Inhibition on Leaf Size in Relay Intercropped Soybean

**DOI:** 10.1371/journal.pone.0098465

**Published:** 2014-06-02

**Authors:** Wanzhuo Gong, Pengfei Qi, Junbo Du, Xin Sun, Xiaoling Wu, Chun Song, Weiguo Liu, Yushan Wu, Xiaobo Yu, Taiwen Yong, Xiaochun Wang, Feng Yang, Yanhong Yan, Wenyu Yang

**Affiliations:** 1 College of Agronomy, Sichuan Agricultural University, Chengdu, China; 2 Key Laboratory of Crop Ecophysiology and Farming System in Southwest China, Ministry of Agriculture, Chengdu, China; 3 Triticeae Research Institute of Sichuan Agricultural University, Chengdu, China; 4 College of Resource and Environment, Sichuan Agricultural University, Chengdu, China; 5 College of Animal Science and Technology, Sichuan Agricultural University, Ya'an, China; Instituto de Biología Molecular y Celular de Plantas, Spain

## Abstract

Multi-species intercropping is a sustainable agricultural practice worldwide used to utilize resources more efficiently. In intercropping systems, short crops often grow under vegetative shade of tall crops. Soybean, one important legume, is often planted in intercropping. However, little is known about the mechanisms of shade inhibition effect on leaf size in soybean leaves at the transcriptome level. We analyzed the transcriptome of shaded soybean leaves via RNA-Seq technology. We found that transcription 1085 genes in mature leaves and 1847 genes in young leaves were significantly affected by shade. Gene ontology analyses showed that expression of genes enriched in polysaccharide metabolism was down-regulated, but genes enriched in auxin stimulus were up-regulated in mature leaves; and genes enriched in cell cycling, DNA-replication were down-regulated in young leaves. These results suggest that the inhibition of higher auxin content and shortage of sugar supply on cell division and cell expansion contribute to smaller and thinner leaf morphology, which highlights potential research targets such as auxin and sugar regulation on leaves for crop adaptation to shade in intercropping.

## Introduction

Multi-species cropping, such as intercropping and agroforestry is a sustainable agricultural practice widely distributed in many developed and developing countries to enhance food security and to use natural resources more efficiently. In intercropping systems, two or more crops are grown simultaneously in the same field during a growing season [1]. During the simultaneous growth of the mixed crops, light is one of the most important limiting factor related to crop yields because of inter-specific competition [2]. Therefore, the effects of shade on understory crops should be considered when trying to increase the productivity of an intercropping system [2].

Shade is ubiquitous in nature, and all plants are shaded to some degree during their lifecycle [3]. Shade conditions are characterized by low levels of photosynthetically active radiation (PAR) and a low ratio of red light to far-red light (R:FR), and both of which are important signaling factors in shade conditions [4], [5]. PAR drives the light reactions of photosynthesis, and is considered to be the primary energy source for photosynthesis in shade-tolerant plants [3], [6], and the R:FR ratio is considered to be a more important light signaling factor.

When exposed to shade, plants can employ two compatible strategies; shade tolerance and shade avoidance [6]. Shade tolerance responses optimize light capture and utilization, including increases of chlorophyll content (Chl), specific leaf area, photosystem II:I ratio, and decrease of chlorophyll a:b ratio (Chl a:b), all of which contribute to carbon gain in the leaf [3], [7], [8]. Shade avoidance responses, which are induced by the signaling factors of low R:FR and low PAR, maximize light capture by positioning the leaves out of the shade via photoreceptor signaling networks [4], [5], [9]–[12].

Leaf is the most abundant photosynthetic organ for carbon gain under low PAR. Many features associated with shade tolerance are attributed to leaf physiology, biochemistry, anatomy and morphology [3]. Leaf area is an important trait for shade tolerance of plants. Generally, plants develop relatively larger leaves at the cost of reduced leaf mass per unit area (LMA), and accumulate higher chlorophyll content per unit mass in shade conditions, these features result in greater opportunities for light capture and harvesting [13]–[16]. At whole plant level, leaf area per whole plant mass is thought to be positively related to shade tolerance, especially for smaller plants [3], but the absolute leaf area is smaller under shade [17]. Leaf area is a basic component of leaf area index for crop production, which contributes to whole light interception of crop canopy [18], [19]. Hence, leaf area is a critical trait for crop to cope with shade. On other side, leaf is also involved in shade avoidance responses. For example, inhibited leaf area is usually observed in shade avoidance response regulated by photoreceptors [17]. As major source plant hormones, gibberellin and indole-3-acetic acid (IAA) contents in leaves are significantly increased by low R:FR ratio [20]. Although R:FR is most closely tied to increased hormone content, both low PAR and low R:FR can influence the levels of endogenous hormones in leaves [20]. Leaf size largely depends on the cell number and cell size. However, leaf area is not simply the sum of cell size and number, in fact, leaf area is under the co-ordination of cell division, cell expansion and overall organ regulation network [21]–[24]. Concerning leaf development, the conversion of a leaf primordium into a mature leaf is usually described as consisting of cell division and cell expansion [21], [22]. Cell division occurs throughout the entire primordium and generates new cells the size of which remains relatively constant and small, after the cessation of cell division further leaf growth is mainly achieved by cell expansion, resulting in a large increase in cell size [22]. Cell division is generally complete when leaves have reached no more than 20% of their final surface area [25]. Previous study found that total leaf area, the number of leaves and individual leaf areas of *Arabidposis* were reduced by the shading treatment [26]. Therefore, it was noteworthy that reduction in leaf number was associated with a reduction in leaf initiation rate and the duration of the phase of leaf production, and these changed leaf expansion dynamics were accompanied by a decrease in epidermal cell number which was partly compensated for by an increase in epidermal cell area [26]. Meanwhile, other study pointed that cell expansion, not cell division, played a major role in the leaf blade growth under shade conditions [17]. In general, previous studies implied that both cell division in early leaf development phase and cell expansion in late leaf development phases finally determine the leaf area. Besides endogenous genetic control, leaf area is also influenced by environmental factors such as light. The shading light promotes petiole elongation and inhibits leaf expansion [17], [27], and sucrose feeding can promote the growth of leaf, irrespective of the light conditions [17]. Plant hormones involve in regulating leaf area. Previous studies found that normal leaf expansion depends on rigorous control of IAA homeostasis, both decreases and increases in the IAA levels in developing leaves led to reductions in leaf expansion, and the IAA content decreased gradually as the young leaves expanded to their full size [28].

Natural environment is more complex. It is difficult to apply reported results conducted under laboratory conditions to problems that occur in complex natural environments or agricultural conditions. Transcriptome studies have made substantial contributions to our understanding of environmental issues and crop improvement in controlled condition. However, only a few studies have analyzed the transcriptome responses of crops to environmental factors under field conditions. Such studies have included analyses of responses to elevated CO_2_ in soybean [29], transcriptome dynamics in response to meteorological factors in rice [30], and critical physiological processes involved in flowering and seed development in rice [31].

Soybean is the fourth most widely cultivated crop worldwide, and is also one of the major crops grown in intercropping systems [32]–[34]. The release of the soybean genome [35] made it possible to analyze the transcriptome responses of intercropped soybean. RNA-Seq has allowed many advances in the characterization and quantification of the transcriptome [36], and has been widely used for gene discovery in soybean [37]–[41]. We observed smaller leaf of soybean grown under shade in intercropping systems. However, our understanding of how crop leaves utilize light in shade in intercropping, and how shade affects leaf development are still limited. The purpose of this study was to characterize the possible mechanisms of shade-induced inhibition on soybean leaf development in intercropping systems at the transcriptome level. This study generated a novel complete data set showing the transcriptome responses to shade in leaves of intercropped soybean in outdoor conditions. Our findings provide valuable data and specific research clues for genetic improvement and physiological analysis of crops cultivated in multi-species cropping systems.

## Materials and Methods

### Plant materials and growth conditions

The experiment was conducted in the farming land of Sichuan Agricultural University, and all the materials were stored in our lab or can be purchased from the market, and no protected species were sampled in our study. Six to ten seeds of the soybean variety Gongxuan 1 were sown in plastic pots (30 cm-diameter) on 19 June 2012. Each pot contained 50 kg sandy soil. Pots were placed under vegetative shade (SH; ∼65% shading level), or full sunlight (FS; control) conditions. In SH treatment, maize was planted on 28 March 2012 with a 50 cm+150 cm wide-narrow row spacing, and the canopy height was about 2.5 m at the sowing time of soybean. Hence, vegetative shade conditions were achieved by placing the pots as pair rows between two wide rows of maize, and the distance from central point of soybean pot to maize row was 50 cm (Figure S5). Field managements were maintained as locally normal production conditions. Since the soybean plants were in pots, there was no below-ground competition from roots. The experiment consisted of three replicates. On the sampling day, light irradiance, temperature, and relative humidity above soybean plants were measured over the course of the day. PAR was monitored by a quantum sensor (LI-190, LI-COR, Lincoln, NE, USA). R:FR was measured by a spectrometer (AvaField-3, Avantes China, Beijing, China) every 1–2 h, and the R:FR spectral range was defined as described by Franklin [42]. Temperature and relative humidity were monitored by a MicroLite data logger (MicroLite-5016, Fourier Systems, Israel). The overall daily light irradiance of SH was about 35% of that in the FS treatment, and the diurnal changes in R:FR, temperature, and relative humidity are shown in Figure S6. Soybeans were thinned to three plants per pot at 20 days after planting. On 7 August (49 days after sowing), six randomly selected plants from six different pots in each replicate were tagged for sampling. Six middle leaflets of the latest fully expanded mature leaves and six middle leaflets of the youngest expanding leaves (leaf length <3 cm) were cut and pooled as mature and young samples, respectively, then wrapped in foil and immediately frozen in liquid nitrogen and stored at −80°C until for RNA extraction.

### Leaf morphology and physiology measurements

For leaf morphology and physiology traits, another six intact plants per replicate were tagged for measurements. Due to the inconvenient of gas exchange measurements and rapid desiccation of young leaves, morphological and physiological measurements were only performed on the mature leaves. For gas exchange measurement, one tagged plants per replicate were used to measure light response curve by the portable photosynthesis system (LI-6400XT, Li-Cor Inc., USA) equipped with 6400-02B Red/Blue LED Light Source. Conditions in the chamber were 25°C for leaf temperature, 60%∼75% for relative humidity, 380 µmol mol^−1^ for CO_2_ concentration. Light responses were then obtained starting from PAR of 2,000 µmol m^−2^ s^−1^ and decreased stepwise to complete darkness. Completed light response curves were fit to a non-rectangular empirical function to estimate maximal assimilation rate (A_max_) [43]. After the gas exchange measurements, mature leaves were sampled and quickly brought to the laboratory for leaf area and chlorophyll content measurements. After the mature middle leaflets were scanned by a flatbed scanner (CanoScan LiDE 200, Canon, Japan), the middle leaflets were enveloped separately and all the plants segments were oven-dried for 72 h to weigh aboveground biomass. Leaf area images were analyzed using ImageJ 1.45 s software. LMA was calculated as leaf mass per unit area. After the oven-dried mature leaves were milled to fine powder, sucrose and starch concentrations were measured as described by Hendrix [44]. For the effects of shade on chlorophyll (total Chl content and Chl a:b) and anatomical traits, mature lateral leaflets were used. Three discs (1 cm-diameter) were punched out and extracted in 80% aqueous acetone. Total Chl content and Chl a:b ratio were determined by spectrophotometric analysis [45]. Two middle segments (5 mm×8 mm) without midrib were cut out and fixed in FAA solution for later paraffin section observation. Total leaf thickness, palisade and spongy mesophyll thickness were quantified by ImageJ 1.45 s. Mean values of each replicate were calculated for data statistics.

### RNA extraction and RNA-sequencing

The cDNA libraries were constructed following the TruSeq™ RNA Sample Preparation Guide (Illumina, San Diego, CA, USA). Briefly, total RNA was isolated with PureLink RNA Mini Kit (Invitrogen, Carlsbad, CA, USA) according to the manufacturer's protocol, and the polyA RNA was isolated using the RNA Purification Beads (Illumina). The mRNAs were fragmented by incubation in Elute, Prime, Fragment Mix at 94 °C for 8 min to obtain 120–200 bp inserts. First strand cDNA was synthesized with SuperScript II Reverse Transcriptase (Invitrogen) using random primer, and Ampure XP beads are used to isolate the double-strand (ds) cDNA synthesized by Second Strand Master Mix. The adapter was ligated to the A-Tailing fragment, and 12 cycles of PCR was performed to enrich those DNA fragments that have adapter molecules on both ends and to amplify the amount of DNA in the library. Purified libraries were quantified by Qubit® 2.0 Fluorometer and validated by Agilent 2100 bioanalyzer to confirm the insert size and calculate the mole concentration. Cluster was generated by cBot with the library diluted to 10 pM and then were sequenced on the Illumina Genome Analyzer IIx for 75 cycles. The library construction and sequencing was performed at Shanghai Biotechnology Corporation.

### RNA-Seq data analysis

The raw reads were cleaned by fastx (Version 0.0.13, http://hannonlab.cshl.edu/fastx_toolkit/) to remove: 1. reads that have more than 50 bases with lower quality than Q20; 2. all the terminal bases which lower than Q20; 3. adaptor sequence; 4. short reads (length <20b). The soybean genome was downloaded from EnsemblPlants (V1.0.15, http://plants.ensembl.org/Glycine_max/Info/Index). Genome mapping was performed using Tophat (version:2.0.4, see parameters in Text S1). After mapping, duplicate reads were removed, and then gene expression analysis of multi-samples was performed using Cuffdiff in Cufflink (V2.0.2, see parameters in Text S1) [46]. The RPKM method was used to normalize the gene transcript levels [47]. Genes with *p*-value<0.01 and |log_2_FC|>1 between SH and FS in mature or young leaves were considered to be significantly differentially transcribed. Differentially transcribed genes in mature or young leaves were selected separately for Gene Ontology (GO) annotation and gene set enrichment analysis. Gene set enrichment analysis of these differentially transcribed genes was performed using AgriGO's singular enrichment analysis (http://bioinfo.cau.edu.cn/agriGO/)[48].

### Quantitative RT-PCR

To verify the transcription levels of genes obtained by RNA-Seq, qRT-PCR analyses were performed for 24 selected genes (Table S3). These selected genes had roles in photosynthesis, carbohydrate metabolism, responses to hormone stimuli, and responses to light stimuli. The total RNA samples used for the qRT-PCR were the same as those used for RNA-Seq. The RNA was treated with RQ1 RNase-free Dnase (Promega, Madison, WI, USA) to digest genomic DNA, and first-strand cDNA was synthesized with a Reverse Transcription System (Promega) according to the manufacturer's instructions. Primer pairs were designed using Primer Premier 5.0 software or as cited in publications [49]. PCR efficiency was calculated using LinRegPCR [50], and the calculated efficiency of each primer pair was 90% to 110%. Each 20 µl qRT-PCR reaction mixture comprised 1 µl 5× dilution cDNA, 10 µl 2×SYBR Select Master Mix (Invitrogen) and 0.4 µl (200 nM) of each primer. The thermal cycling conditions were according to the manufacturer's protocol, as follows: 50°C for 2 min, 95 °C for 2 min, followed by 40 cycles of 95°C for 15 s, and 60°C for 60 s. Melting curve analysis was performed at the end of the PCR amplification over the range of 60°C to 95°C, increasing the temperature stepwise by 0.5°C every 10 s. The cycle threshold (Ct) values were automatically calculated. The relative transcript levels of genes were calculated using the 2^−ΔΔC^_T_ method [51]. A commonly used reference gene for soybean, ACT11 (Glyma18g52780) was used as control [52], and qRT-PCR was performed with three technical replicates for each sample on the same plate.

### Data statistics

Differences between SH and FS treatments were analyzed by ANOVA in SPSS software (SPSS, Chicago, USA). All measured and calculated features were analyzed as dependent variable; cropping treatments was analyzed as fixed factors. Correlation analysis between results of qRT-PCR and RNA-Seq was analyzed by SPSS software, Spearman coefficient was used to estimate the relationship.

## Results

### Shade inhibited leaf size and growth of soybean

To investigate the effect of shade on leaf morphological traits, we measured leaf area, LMA, and leaf anatomical traits for soybean plants grown in the shade and in full sunlight. (Table 1). There were significant differences in leaf area, LMA and anatomical thickness. The decreased leaf area, LMA, and total leaf thickness confirmed that the leaves of plants grown under long-term shade conditions became smaller and thinner as predicted [17], [53]. Meanwhile, we also observed reduced A_max_, sucrose and starch contents in shade grown soybean (Table 1). Therefore, the smaller and thinner leaves led to a lower light interception and photosynthetic capacity, resulting in reduced supplies of photosynthetic products. Ultimately, the soybean biomass declined in shade.

**Table 1:** Leaf morphological and physiological traits of soybean under shade and full sunlight conditions a.

Trait	Light condition	
	SH	FS
Leaf area (cm^2^)	40.91±2.76^b^	69.45±4.48^a^
LMA (g m^−2^)	20.33±0.58 ^b^	38.09±0.64 a
Palisade thickness (µm)	29.33±1.34 ^b^	55.97±1.22 a
Spongy thickness (µm)	18.11±1.27 ^b^	33.41±0.53 a
Total leaf thickness(µm)	61.60±3.04 ^b^	109.66±1.31 a
Chl content (mg g^−1^ DM)	12.45±0.55 a	9.38±0.50 ^b^
Chl a:b	2.468±0.043 ^b^	2.746±0.040 a
A_max_ (mg g^−1^ DM)	17.64±1.15 ^b^	26.67±1.90 a
Sucrose content (mg g^−1^ DM)	65.25±10.92 ^b^	122.18±9.98 a
Starch content (mg g^−1^ DM)	18.7±2.4 ^b^	40.5±15.7 a
Biomass (g plant^−1^)	1.37±0.10 ^b^	5.60±0.46 a

a Morphological and physiological traits were measured using mature leaves. Leaf area represents the middle leaflet of the latest full expanded leaves. Different letters in each row indicate a significant difference with the method of one-way ANOVA between shade and sunlight conditions (*p*<0.05).

In plants, most chlorophyll combines with pigment-binding proteins to form light-harvesting complexes in the thylakoids [7], [54]. Therefore, the higher chlorophyll content per leaf mass in shaded soybean leaves suggested that there would be more pigment-binding proteins. Since the light-harvesting complex of photosystem II contains most chlorophyll b, the reduced Chl a:b under shade conditions could be because there were more photosystem II light-harvesting complexes. However, the chlorophyll compensation could not negate the decreases of photosynthetic products, consistent with the result of significant lower biomass plants. Therefore, these results confirmed that the shade environment inhibited leaf size and biomass of soybean in intercropping.

### Overall evaluation and screening of differentially transcribed genes

The Illumina 100-bp pair-end sequencing generated 27.7 to 43.8 million raw reads and 2.8 to 4.4 gigabyte bases (Table 2) for each of the 12 libraries (mature and young leaves grown under shade or full-sunlight conditions, with three replicates). After trimming bad reads from raw data, 23.9 to 37.8 million effective reads were generated. The effective reads ratios were 85.9% to 95.1%. Genome mapping by Tophat successfully aligned 22.7 to 35.8 million reads to the soybean reference genome.

**Table 2:** Statistics of Illumina reads and comparison to soybean genome.

Leaf type	Condition	Replicate	Number of raw reads	Number of effective reads	Effective reads ratio (%)	Mapped reads	Mapping ratio (%)
Mature	SH	1	33,666,918	28,949,884	86.0	27,530,784	95.1
		2	27,701,676	23,918,686	86.3	22,673,102	94.8
		3	31,403,304	29,874,856	95.1	28,221,662	94.5
	FS	1	29,249,598	25,319,346	86.6	24,057,947	95.0
		2	36,774,498	31,949,380	86.9	30,298,226	94.8
		3	35,608,976	30,778,360	86.4	29,300,192	95.2
Young	SH	1	38,281,788	32,888,660	85.9	30,984,388	94.2
		2	43,640,128	37,497,842	85.9	35,369,215	94.3
		3	31,009,698	26,736,094	86.2	25,308,884	94.7
	FS	1	36,221,884	31,153,954	86.0	29,452,749	94.5
		2	43,837,540	37,796,202	86.2	35,813,139	94.8
		3	34,012,220	29,452,586	86.6	27,974,155	95.0

By using the RKPM values of three replicates between shade and full sunlight conditions with *p*-value<0.01, 1085 and 1847 genes were identified as being differentially transcribed in response to shade in mature and young leaves, respectively. In mature leaves, 348 genes were uniquely up-regulated, and 610 genes were uniquely down-regulated in SH as compared with those in FS conditions. In young leaves, 599 genes and 1121 genes were uniquely up- and down-regulated in SH conditions, respectively. Forty-one genes were up-regulated, and 86 genes were down-regulated in both mature and young leaves. A list of differentially transcribed genes is shown in Table S1 and Table S2. These differentially transcribed genes in mature or young leaves were used for gene annotation and functional analysis.

### Validation of transcripts by qRT-PCR

**Figure 1:**
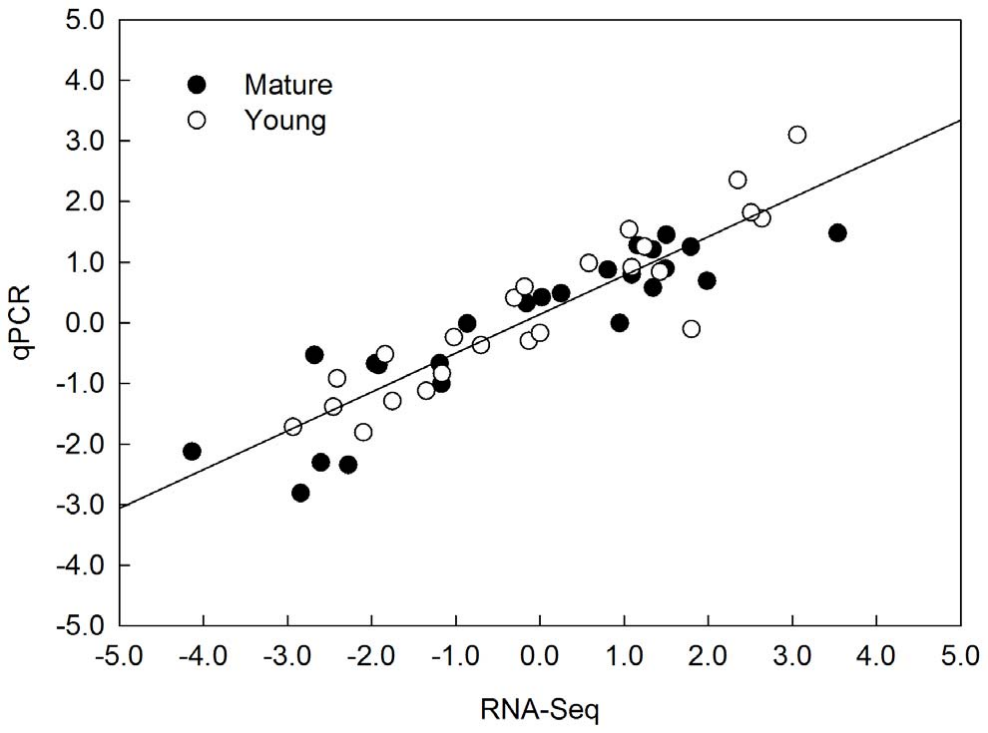
Correlation between the RNA-Seq and qRT-PCR data on identified differentially transcribed genes. Data are log_2_FC values from RNA-Seq and qPCR analyses. 24 genes were randomly selected for comparison between RNA-Seq and qRT-PCR. Filled circles represent data in mature leaves, open circles represent data in young leaves. The overall Spearman correlation of mature and young leaves is 0.910 (*p*<0.001).

To verify the reliability of Illumina sequencing, 24 genes were selected for qRT-PCR assays. Detailed information about candidate genes and primer pairs is provided in Table S3. For the 24 genes analyzed, the qRT-PCR results were generally consistent with the RPKM values determined by RNA-Seq (Figure 1). Correlation analysis showed a 0.910 (*p*<0.001) Spearman coefficient between RNA-Seq and qPCR data. This confirmed the reliability and accuracy of the RNA-Seq technology used in this study.

### GO annotation and functional analysis of transcripts

**Figure 2:**
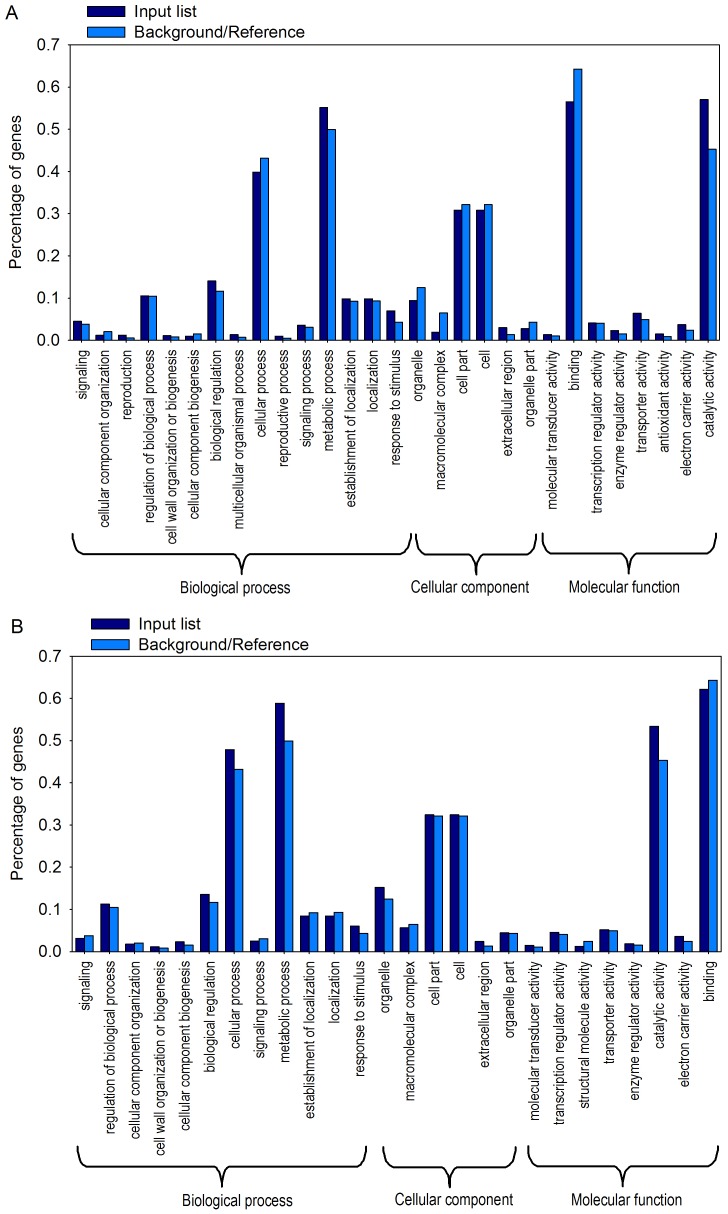
Functional categorization of genes showing differential transcription patterns in response to shade in mature and young leaves. Functional categorization was performed using AgriGO. (A) Functional categorization of differentially transcribed genes in mature leaves; (B) Functional categorization of differentially transcribed genes in young leaves. Blue and green columns represent query (input) and reference lists, respectively.

After screening differentially transcribed genes, the GO IDs were obtained from Ensemble Plant. The differentially transcribed genes in mature or young leaves were used for GO analysis. Gene set enrichment analysis was performed at the AgriGO website. The input genes were classified into biological process, cellular component, and molecular function, and further respective subsets. There were 733 and 1176 annotated differentially transcribed genes in mature and young leaves, respectively. Enrichment analysis showed that 66 and 83 nodes were significant in mature and young leaves, respectively. Further clustering of differentially transcribed genes determined their biological functions (Figure 2). In mature leaves, genes were categorized into 15 subsets within biological process, 6 subsets within cellular component, and 8 subsets within molecular function. In young leaves, genes were categorized into 12 subsets within biological process, 6 subsets within cellular component, and 8 subsets within molecular function.

**Figure 3:**
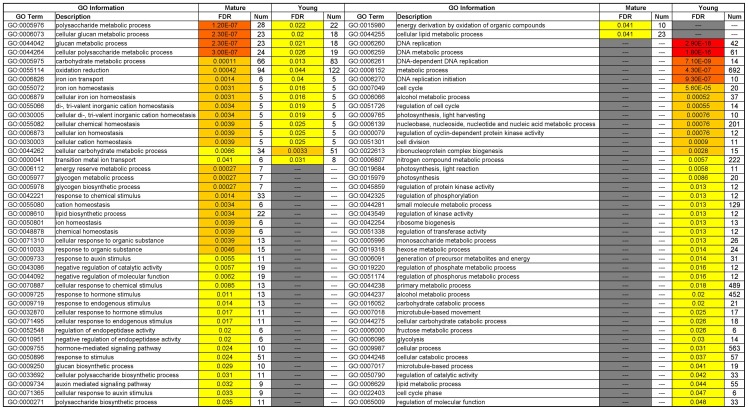
Comparison of significant GO terms within biological process from singular enrichment analyses between mature and young leaves. Significant (*p*<0.05) GO terms within biological process with FDR<0.05 in mature and young leaves are listed. Num column represents the number of genes enriched in each GO term. Darker colors indicate higher significance levels.

To better understand responses of soybean leaves to shade, GO hierarchy images of individual biological process, cellular component, and molecular function were generated. In these visualized GO hierarchy images, nodes with FDR<0.05 are marked with color (Figure S1 and Figure S2). The significant GO terms within biological process are listed in Figure 3 (GO terms within cellular component or molecular function are shown in Figure S3 and Figure S4). There were 16 significant GO terms common to mature and young leaves, 29 for mature leaves, and 41 for young leaves. Generally, this analysis showed that the most significant GO terms (FDR<0.01) were related to polysaccharide metabolism and auxin stimulus in mature leaves, and were related to DNA replication, cell cycle and photosynthesis in young leaves. Thus, the differentially transcribed genes enriched in polysaccharide metabolic process (GO:0005976), response to auxin stimulus (GO:0009733), photosynthesis (GO:0015979), cell cycle (GO:0007049) and DNA replication (GO:0006260) are listed in Table S4 and were used for the following analyses.

### Effect of shade on cell cycle, DNA replication and photosynthesis in young leaves

There were 20 and 42 differentially transcribed genes enriched in cell cycle (GO:0007049) and DNA replication (GO:0006260) in young leaves, respectively. Among them, there were 13 genes encoding cyclins, 10 genes encoding minichromosome maintenance proteins, 9 genes encoding DNA-directed DNA polymerases (Table S4). Furthermore, nearly all of them were down-regulated in shade condition.

Meanwhile, twenty genes related to photosynthesis were differentially transcribed in shade conditions. Surprisingly, all of these genes were up-regulated by shade. Among these genes, 10 encoded chlorophyll a/b-binding proteins, 4 encoded photosystem II reaction center complex subunits, 4 encoded fructose-1,6-bisphosphatase (FBPase), 1 encoded a photosystem I reaction center complex subunit, and 1 encoded a cytochrome b6f complex subunit.

### Effect of shade on polysaccharide metabolism and auxin-stimulus in young leaves

There were 28 and 22 differentially transcribed genes enriched in polysaccharide metabolic process (GO:0005976) in mature and young leaves, respectively. Taken together, 48 genes enriched in this GO term were found (Table S4). Specifically, 29 genes were found involved in cell wall and starch biosynthesis. For example, seventeen genes encoding xyloglucan endo-transglycosylases (XET) were down-regulated in mature or young leaves in the shade. And six cellulose synthase genes were differentially transcribed in shade. In addition, genes encoding reversibly glycosylated polypeptides (RGPs) and rhamnose biosynthetic enzyme (RBE) were also found down-regulated in the shade. Besides cell wall-related genes, we also found some starch related genes were differentially transcribed by shade. For instance, four ADP-glucose pyrophosphorylase (AGPase) genes, two granule-bound starch synthase (GBSS) genes and two starch-branching enzyme (SBE) genes were down-regulated in mature or young leaves in the shade.

Genes associated with the response to auxin stimulus were also interesting findings in this study. In mature leaves, 11 genes involved in auxin stimulus were differentially transcribed in shaded soybean. Among them were 9 genes encoding IAA-inducible proteins, and all of the nine genes were up-regulated in mature or young leaves in the shade.

## Discussion

In shade condition, plant leaves contain more chlorophyll for high efficient capture of light. The increased chlorophyll content (Table 1) and transcript abundances of chlorophyll a/b-binding proteins genes showed that soybean increased transcriptions of light reaction-related genes to acclimate to the low-light conditions for optimizing capture of the limited light resource [3], [7], [54]. But these benefits could not negate the reduction of leaf area and photosynthetic capacity in shade condition, and finally resulted in significant reduction on biomass (Table 1). Smaller and thinner leaves in this study confirmed the shade effects on leaf size of soybean. It has been long-standing known that thinner leaves with a low LMA is a survival strategy that increases opportunities to relatively intercept light [3], [15], [55]. In terms of ecophysiological benefits of thicker sun leaves, it has been suggested that sun leaves have sufficient mesophyll surfaces in palisade tissue occupied by chloroplasts to secure the area for CO_2_ dissolution and transport [15]. Thus, thinner palisade tissue have disadvantages in terms of photosynthetic capacity when light irradiance increases [56], consequently, photosynthetic products supply reduces in thinner leaves. Our measurements on sucrose and starch contents confirmed the reductions of photosynthetic product supplies. For leaf area, it has been reported that shading light inhibited leaf expansion [17], [27], which consequently resulted in the reduction of the light interception. Our observation coincided with these studies and confirmed that soybean leaves were inhibited by shade in intercropping.

### Inhibition on cell division reduced cell number in leaves

The RNA-Seq analysis suggested the possible mechanisms of shade inhibition on leaf size. In this study, we found some down-regulated cyclins, minichromosome maintenance proteins and DNA polymerases in young leaves. Cyclins are eukaryotic proteins that play an active role in controlling nuclear cell division cycles. Minichromosome maintenance proteins are DNA-dependent ATPases required for the initiation of eukaryotic DNA replication, and DNA-directed DNA polymerase accurate replication in the life cycle of a cell. Taken together, the results of down-regulated of genes involved in cell cycle and DNA replication suggested the inhibition of shade on cell cycling in soybean young leaves. Earlier studies showed that cell cyclin related to cell division during leaf development [57], and the dicot leaf area and cell numbers were reduced in shade condition [58]. Leaf area depends on cell number and cell size, especially correlated with cell number [21], [22]. The cell growth can be best described as the succession of five overlapping and interconnected phases: an initiation phase, a general cell division phase, a transition phase, a cell expansion phase, and a meristemoid division phase [22]. Previous study had pointed out that the reduction in individual leaf area grown in shading treatment were accompanied by a decrease in epidermal cell number [26], and the cell division is generally complete when leaves have reached no more than 20% of their final mature area [25]. Therefore, our results suggested that shading condition inhibited the cell division in young leaves, which resulted in the fewer cell numbers and ultimately inhibited leaf area in mature leaves.

### Limited cell wall synthesis inhibited cell expansion

Plant cell walls are mainly composed of polysaccharides, both primary and secondary cell walls contain cellulose and hemicellulose. Xyloglucan is one of the four main classes of hemicellulose [59]. In *Arabidopsis*, XET is a cell wall protein that mediates the exporting of nascent xyloglucan chains to the cell wall matrix and incorporating into the existing xyloglucans [60]. The expression of XET was strongly correlated with tissue expansion, and was regulated by sucrose, hormones, and environmental stimuli [61]. Recently, it was reported that XET in the petiole can be regulated by light quality in shade conditions [62]. Cellulose synthase subunits form a multi-enzyme complex [63] that biosynthesizes cellulose, the core component of cell walls. Several genes encoding subunits of cellulose synthase have been identified in *Arabidopsis* [64], while the environmental regulatory mechanisms that control these genes are unclear. The expression levels of cellulose synthase genes in this study suggested complex regulation of cellulose synthesis in soybean leaves under shade conditions. RGPs are associated with polysaccharide biosynthesis, and may function in cell wall and/or starch synthesis [65]. The down-regulation of RGP genes suggested that the biosynthesis of leaf cell walls was repressed in the shade. Rhamnose is an important constituent of pectic polysaccharides, another major component of cell walls. Overexpression of the *RHM1* gene in *Arabidopsis* increased the rhamnose content by as much as 40% in the leaf cell wall, compared with that of wild type [66]. The down-regulation of RBE genes in the shade suggested that the rhamnose content in the cell wall was reduced under these conditions. Taken together, it can be supposed that cell wall biosynthesis were decreased in shade, but molecular mechanism needs further investigation in future.

Starch is a major photosynthetic products stored in chloroplast in daytime and degraded in night. In this study, starch biosynthesis-related genes encoding AGPase, GBSS, and SBE were generally down-regulated in the shade. Previous studies found the expression of AGPase and GBSS can be controlled by sucrose status [67], [68], and the exogenous application of sugars can induce AGPase activity [69]. In addition, SBE gene expression was induced 24 h after rice leaves were transferred from low to high light conditions [70]. Therefore, the down-regulated starch related genes and starch content suggested that there were low levels of photosynthetic products were contained in leaves in shade. Combined with down-regulation of cell wall synthesis-related genes in our study, we supposed that polysaccharide metabolisms, including cell wall and starch, were inhibited by shade.

Leaves are important sources for auxin synthesis [28]; conversely, leaf development [71], growth, expansion [72], and longevity [73] are controlled by auxin. Both components of shade light, low PAR and a low R:FR ratio, can affect the IAA levels in leaves [20], [74]. Indeed, low R:FR triggers reciprocal control, leading to an increase in IAA production[75], and rapid auxin biosynthesis is required to initiate the multiple changes in shoot shape associated with shade avoidance [76]. Many critical genes involved in IAA regulation under shade have identified on model plant [12]. Recent reports showed that a low R:FR ratio significantly increased the IAA content in leaves in sunflower [20]. Besides a low R:FR ratio, low PAR also alter the auxin signals [77], an increase in auxin activity (IAA-mediated gene expression) was detected in rosette leaves of *Arabidopsis* grown under low PAR. Hence, it seems that the two components of shade light, low PAR and low R:FR, may increase IAA contents in the leaves. IAA is a rigorous regulator in leaf expansion [28], it has been reported that cell expansion in leaves is promoted by auxin only at lower concentrations, and higher auxin concentration suppresses leaf expansion [72], and smaller leaves contained high levels of auxin [78]. Our observation of smaller soybean leaves in shade conditions suggested that higher IAA contents may be contained in mature leaves, and inhibited leaf growth. Another issue should be considered was the changed leaf expansion dynamics. Previous study reported the reduction in leaf expansion rate and an increase in the duration of leaf expansion in shade condition[26]. In addition, IAA content decreased gradually when the young leaves expanded to their full size, and this decrease was accompanied by a clear shift in both pool size and IAA synthesis capacity [28]. Taken together, the rate of IAA decrease during leaf development might become slower in shade condition. Therefore, the biosynthesis and transport of IAA influenced by the interaction between low PAR and low R:FR in leaves, and the dynamics of leaf growth and IAA homeostasis might be potential research targets for improving performance of soybean in intercropping systems.

Plant growth requires irreversible enlargement of cells, and the expansion and elongation of plant cells require not only cell wall loosening, but also deposition of new cell wall materials [60], [79]. Another interesting issue is the opposite direction of the up-regulated IAA-inducible proteins and down-regulated XET. It has been reviewed that both auxin and XET involve in the cell wall loosen and expansion process during cell expansion [60]. These results suggested that auxin might have induced cell wall loosening, but the paucity of materials for synthesis of cell wall polysaccharides might have restricted cell enlargement in shade. Previous sugar feeding experiment on shade grown *Arabidopsis* showed that sucrose feeding could increase leaf area[17]. Thus, it can be suggested that cell expansion process in leaves grown in shade might also be inhibited as the results of lacking synthesis of cell wall matrix. The interaction of auxin and sugar supply on leaf expansion needs further study in the near future.

In summary, soybean leaf became smaller and thinner in shade intercropping. 1085 and 1847 genes were found affected in mature and young leaves by comprehensive analysis of the transcriptome of soybean leaves in response to shade. Gene ontology analyses showed that genes modulating in polysaccharide metabolism were down-regulated and genes induced in auxin stimulus were up-regulated in mature leaves; and genes regulating in cell cycling, DNA-replication were down-regulated in young leaves. RNA-Seq results suggested the inhibition of higher auxin content and shortage of sugar supply on cell division and cell expansion in smaller and thinner leaf morphology. Thus, the interaction of auxin and sugar regulation in leaf expansion might be research targets for soybean genotypic improvement with aims for better adaptation to intercropping.

## Supporting Information

Figure S1 **GO hierarchy image for biological process, based on gene set enrichment analysis in mature leaves.** The GO hieratical image containing all statistically significant terms. Darker colors indicate higher significance levels.
(TIF)

Figure S2 **GO hierarchy image for biological process, based on gene set enrichment analysis in young leaves.** The GO hieratical image containing all statistically significant terms. Darker colors indicate higher significance levels.
(TIF)

Figure S3 **Comparison of significant GO terms within cellular component, based on singular enrichment analyses between mature and young leaves.** Significant (*p*<0.05) GO terms within biological process with FDR<0.05 in mature and young leaves are listed. Num column represents the number of genes enriched in each GO term. Darker colors indicate higher significance levels.
(TIF)

Figure S4 **Comparison of significant GO terms within molecular function, based on singular enrichment analyses between mature and young leaves.** Significant (*p*<0.05) GO terms within biological process with FDR<0.05 in mature and young leaves are listed. Num column represents the number of genes enriched in each GO term. Darker colors indicate higher significance levels.
(TIF)

Figure S5 **Schematic diagram of shade (SH) treatments.** Black lines represent the maize rows. Maize was planted on 28 March 2012 with a 50 cm+150 cm wide-narrow row spacing. White circles represent soybean pot. Soybean was planted on 19 June 2012, and the distance from central point of soybean pot to maize row was 50 cm.
(TIF)

Figure S6 **Microclimate data under shade (SH) and full sunlight (FS) treatments on the sampling day.** Light characteristics, temperature, and relative humidity above soybean plants were measured over the course of the day. PAR was monitored using a quantum sensor (LI-190, LI-COR). R:FR was measured every 1–2 h using a spectrometer (Avantes), and the R:FR spectral range was defined as described by Franklin [42]. Temperature and relative humidity were monitored by a MicroLite data logger (Fourier).
(TIF)

Table S1 Differentially transcribed genes (log_2_FC>1.0, *p*<0.01) in mature leaves between shade (SH) and full sunlight (FS) conditions.
(XLS)

Table S2 Differentially transcribed genes (log_2_FC>1.0, *p*<0.01) in young leaves between shade (SH) and full sunlight (FS) conditions.
(XLS)

Table S3 Primer sequences of genes used to validate RNA-seq results by qRT-PCR.
(XLS)

Table S4 Differentially transcribed genes between mature and young leaves.
(XLS)

Text S1 **Parameters used in RNA-Seq analysis.**
(TXT)
