# Supplementary figures and images for: Transcriptome Analysis of Shade-Induced Inhibition on Leaf Size in Relay Intercropped Soybean
Source: PLoS One. 2014 Jun 2;9(6):e98465. doi: 10.1371/journal.pone.0098465 (PMC4041726; doi:10.1371/journal.pone.0098465)

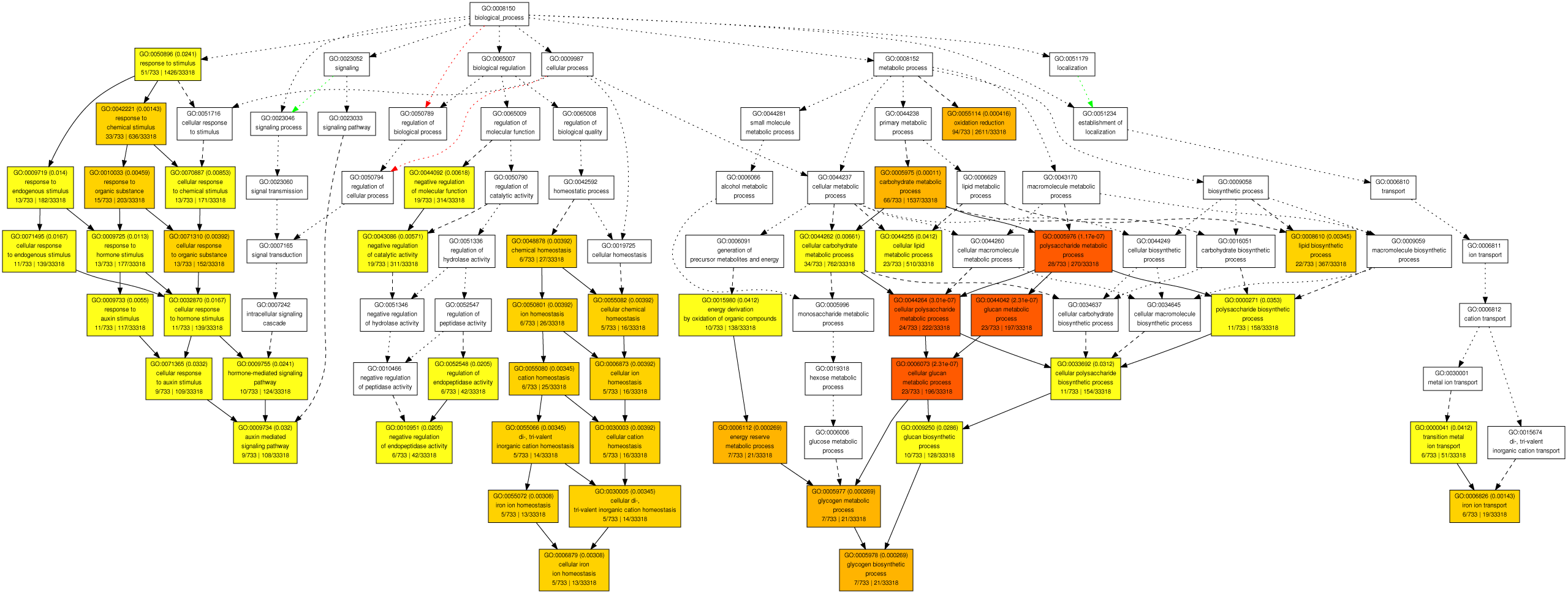

Supplement: Figure S1 — GO hierarchy image for biological process, based on gene set enrichment analysis in mature leaves. The GO hieratical image containing all statistically significant terms. Darker colors indicate higher significance levels. (TIF) [file pone.0098465.s001.tif]

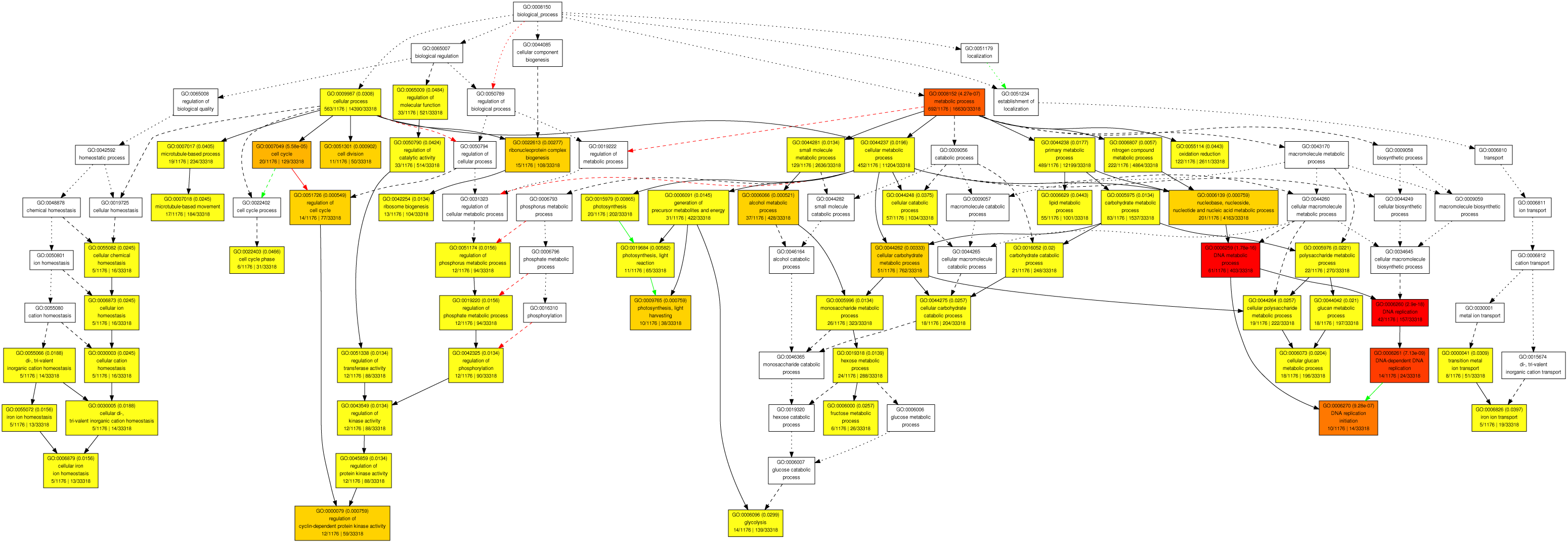

Supplement: Figure S2 — GO hierarchy image for biological process, based on gene set enrichment analysis in young leaves. The GO hieratical image containing all statistically significant terms. Darker colors indicate higher significance levels. (TIF) [file pone.0098465.s002.tif]

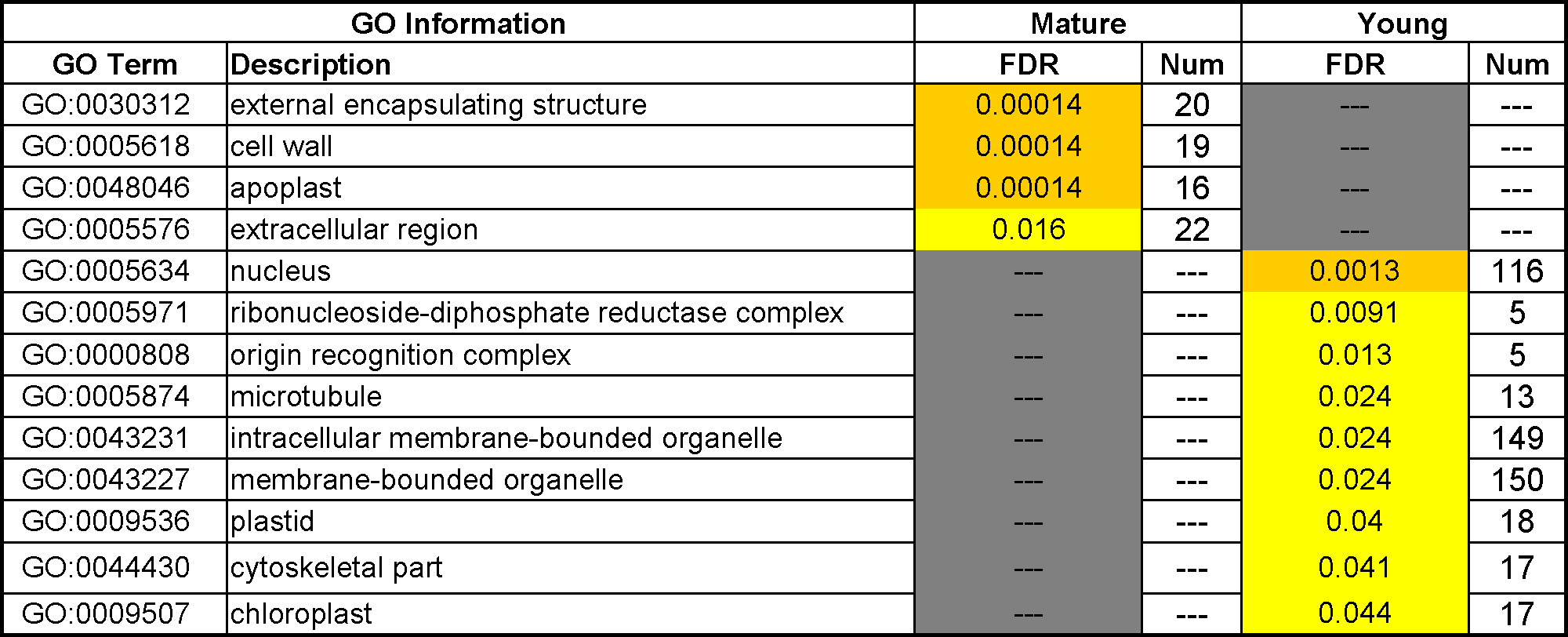

Supplement: Figure S3 — Comparison of significant GO terms within cellular component, based on singular enrichment analyses between mature and young leaves. Significant (p<0.05) GO terms within biological process with FDR<0.05 in mature and young leaves are listed. Num column represents the number of genes enriched in each GO term. Darker colors indicate higher significance levels. (TIF) [file pone.0098465.s003.tif]

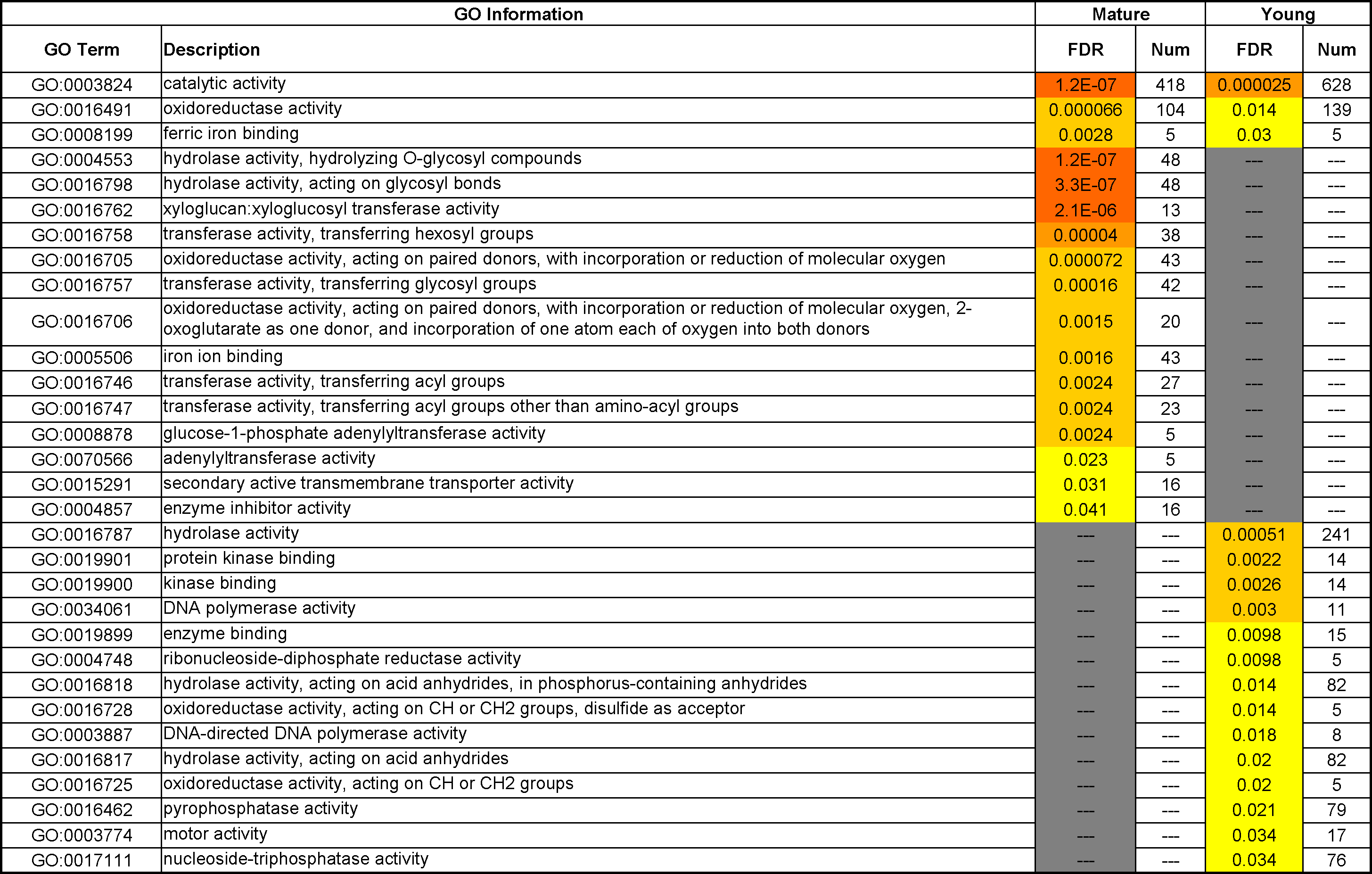

Supplement: Figure S4 — Comparison of significant GO terms within molecular function, based on singular enrichment analyses between mature and young leaves. Significant (p<0.05) GO terms within biological process with FDR<0.05 in mature and young leaves are listed. Num column represents the number of genes enriched in each GO term. Darker colors indicate higher significance levels. (TIF) [file pone.0098465.s004.tif]

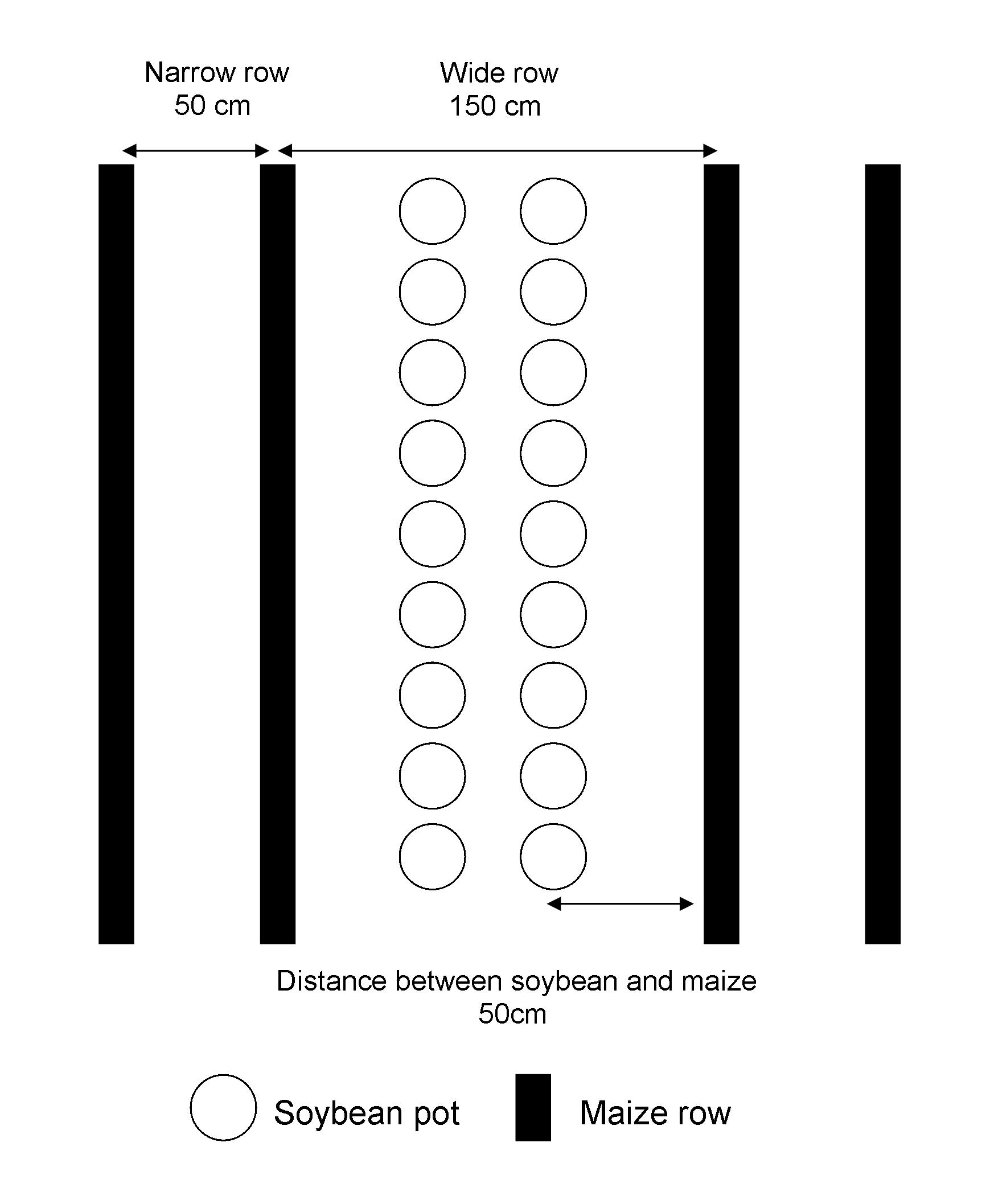

Supplement: Figure S5 — Schematic diagram of shade (SH) treatments. Black lines represent the maize rows. Maize was planted on 28 March 2012 with a 50 cm+150 cm wide-narrow row spacing. White circles represent soybean pot. Soybean was planted on 19 June 2012, and the distance from central point of soybean pot to maize row was 50 cm. (TIF) [file pone.0098465.s005.tif]
